# (*Z*)-Methyl 4-({3-[(2,5-dioxoimidazolidin-4-yl­idene)meth­yl]-1*H*-indol-1-yl}meth­yl)benzoate

**DOI:** 10.1107/S1600536808041020

**Published:** 2008-12-10

**Authors:** Narsimha Reddy Penthala, Thirupathi Reddy Yerram Reddy, Sean Parkin, Peter A. Crooks

**Affiliations:** a Department of Pharmaceutical Sciences, College of Pharmacy, University of Kentucky, Lexington, KY 40536, USA; b Department of Chemistry, University of Kentucky, Lexington, KY 40506, USA

## Abstract

In the title compound, C_21_H_17_N_3_O_4_, pairs of mol­ecules form a planar[maximum deviation 0.0566 (9) Å] centrosymmetric imidazole dimer *via* two N—H⋯O hydrogen bonds. These dimeric units are linked by further N—H⋯O hydrogen bonds between the ester carbonyl group and the imidazolidine ring, formiing chains parallel to the *c*-axis direction. In addition, there are π–π stacking inter­actions between the planar imidazole pairs, with an inter­planar spacing of 3.301 (2) Å. There is a double bond with *Z* geometry connecting the imidazolidine and indole units.

## Related literature

For general background to the radiosensitization activity of (*Z*)-2-(*N*-benzyl­indol-3-ylmethyl­ene)quinuclidin-3-one and (*Z*)-(±)-2-(*N*-benzyl­indol-3-ylmethyl­ene)quinuclidin-3-ol derivatives, see: Sekhar *et al.* (2003 ▶); Sonar *et al.*, (2007 ▶). For related structures, see: Mason *et al.* (2003 ▶); Zarza *et al.* (1988 ▶).
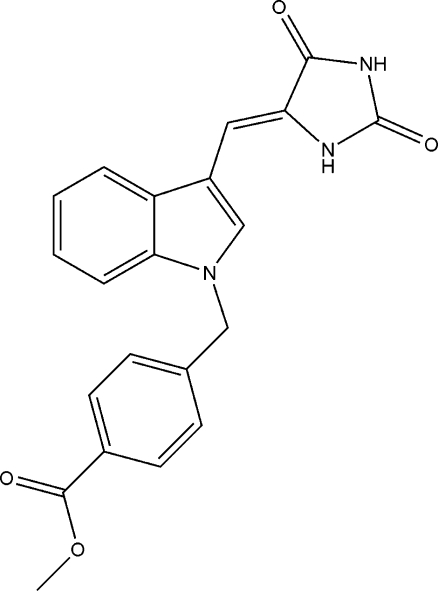


## Experimental

### 

#### Crystal data

C_21_H_17_N_3_O_4_
*M* *_r_* = 375.38
Triclinic, 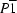

*a* = 7.6390 (1) Å
*b* = 8.0013 (1) Å
*c* = 15.0405 (3) Å
α = 91.9853 (9)°
β = 96.2291 (9)°
γ = 104.4242 (9)°
*V* = 883.25 (2) Å^3^
*Z* = 2
Mo *K*α radiation
μ = 0.10 mm^−1^
*T* = 90.0 (2) K
0.25 × 0.22 × 0.15 mm

#### Data collection

Nonius KappaCCD diffractometer
Absorption correction: multi-scan (*SCALEPACK*; Otwinowski & Minor, 1997 ▶) *T*_min_ = 0.976, *T*_max_ = 0.985
19513 measured reflections
3997 independent reflections
3595 reflections with *I* > 2σ(*I*)
*R*_int_ = 0.015

#### Refinement

*R*[*F* ^2^ > 2σ(*F* ^2^)] = 0.043
*wR*(*F* ^2^) = 0.127
*S* = 1.07
3997 reflections
255 parameters
H-atom parameters constrained
Δρ_max_ = 0.48 e Å^−3^
Δρ_min_ = −0.34 e Å^−3^

### 

Data collection: *COLLECT* (Nonius, 1998 ▶); cell refinement: *SCALEPACK* (Otwinowski & Minor, 1997 ▶); data reduction: *DENZO-SMN* (Otwinowski & Minor, 1997 ▶); program(s) used to solve structure: *SHELXS97* (Sheldrick, 2008 ▶); program(s) used to refine structure: *SHELXL97* (Sheldrick, 2008 ▶); molecular graphics: *XP* in *SHELXTL* (Sheldrick, 2008 ▶); software used to prepare material for publication: *SHELXL97* and local procedures.

## Supplementary Material

Crystal structure: contains datablocks global, I. DOI: 10.1107/S1600536808041020/fj2166sup1.cif

Structure factors: contains datablocks I. DOI: 10.1107/S1600536808041020/fj2166Isup2.hkl

Additional supplementary materials:  crystallographic information; 3D view; checkCIF report

## Figures and Tables

**Table 1:** Hydrogen-bond geometry (Å, °)

*D*—H⋯*A*	*D*—H	H⋯*A*	*D*⋯*A*	*D*—H⋯*A*
N11—H11⋯O12^i^	0.88	2.11	2.9658 (15)	163
N13—H13⋯O22^ii^	0.88	2.29	2.9699 (15)	134

Symmetry codes: (i) 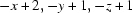; (ii) 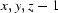.

**Fig. 1.**
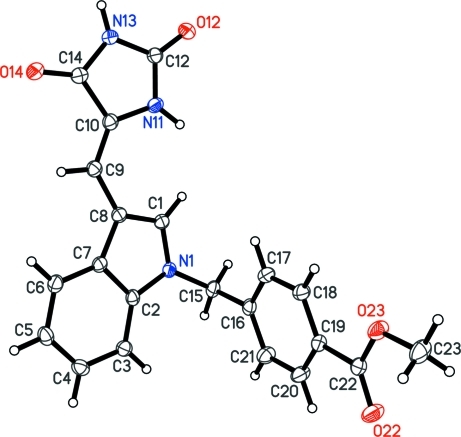
A view of the molecule with the atom numbering scheme. Displacement ellipsoids are drawn at the 50% probability level.

## supplementary crystallographic information

### Comment

In continuation of our work on the radiosensitization activity of (*Z*)-2-(*N*-benzylindol-3-ylmethylene)quinuclidin-3-one and (*Z*)-(±)-2-(*N*-benzylindol-3-ylmethylene) quinuclidin-3-ol derivatives (Sekhar *et al.*, 2003; Sonar *et al.*, 2007), we have undertaken the design, synthesis and structural analysis of a series of (*N*-benzylindol-3-ylmethylene)imidazolidine-2,4-dione analogs with different substituents on both indole moiety and on the benzene ring of the *N*-benzyl group. The primary goal for X-ray analysis of the title compound is to confirm the double-bond geometry and to obtain detailed information on the structural conformation of the molecule. This information will be useful in structure-activity relationship (SAR) analysis. The title compound was prepared by the reaction of methyl 4-((3-formyl-1*H*-indol-1-yl)methyl)benzoate with imidazolidine-2,4-dione in the presence of ammonium acetate in acetic acid at 391 K. The compound was crystallized from a mixture of methanol and ethylacetate. The molecular structure and the atom-numbering scheme are shown in Fig.1. The indole ring is planar with bond distances and angles comparable with those previously reported for other indole derivatives (Mason *et al.*, 2003; Zarza, *et al.*, 1988). The X-ray studies revealed that the title compound is the *Z* isomer. The C8—C9 bond is in a *transoid* geometry with respect to the C10—C14 bond. The olefinic bond (C9=C10) has a planar atomic arrangement, since the r.m.s. deviation from the mean plane passing through atoms C1, C8, C9, N11 is 0.0349 (6) Å. Deviations from ideal geometry are observed in the bond angles around atoms C9, C10 and N11 (130.48 (12)°) due to repulsion between the indole ring C1 hydrogen and imidazolidine ring N11 hydrogen. The imidazolidine ring, which makes a dihedral angle of 10.03 (7)° with the adjacent aromatic ring, presents very small distortions around atoms N11, C12, N13 and C14.

Significant intermolecular hydrogen-bonding interactions are found between N(11)—H(11)···O(12) and N(13)—H(13)···O(22), and molecules are linked into chains by N—H···O hydrogen bonding.

### Experimental

A mixture of methyl 4-((3-formyl-1*H*-indol-1-yl)methyl)benzoate (0.5 g, 1.70 mmol), imidazolidine-2,4-dione (0.18 g, 1.80 mmol) and ammonium acetate (0.132 g, 1.71 mmol) was stirred in acetic acid (5 ml) at 391 K for 8 hrs. The reaction mixture was cooled to room temperature and the yellow solid that separated was collected by filtration, washed with cold water and dried to afford the crude product. Crystallization from methanol and ethyl acetate (1:1) afforded a yellow crystalline product of (*Z*)Methyl-4-((3-((2,5- dioxoimidazolidin-4-ylidene)methyl)-1*H*-indol-1-yl)methyl)benzoate that was suitable for X-ray analysis. ^1^H NMR (DMSO d_6_): δ 3.81 (*s*, 3H), 5.54 (*s*, 2H), 6.74 (*s*, 1H), 7.15–7.23 (*m*, 2H), 7.40–7.42 (*d*, 2H), 7.50–7.52 (*d*, 1H), 7.79–7.81 (*d*, 1H), 7.91–7.93 (*d*, 2H), 8.32 (*s*, 1H), 10.15 (*bs*, 1H), 11.06 (*bs*, 1H); ^13^C NMR (DMSO d_6_): δ 49.52, 52.2, 101.44, 109.26, 111.26, 119.22, 121.35, 123.44, 124.01, 127.29, 128.95, 129.35, 131.23, 136.28, 142.64, 155.93, 165.85, 167.12.

### Refinement

H atoms were found in difference Fourier maps and subsequently placed in idealized positions with constrained distances of 0.98 Å (RCH_3_), 0.99 Å (*R*_2_CH_2_), 0.95 Å (C_Ar_H), 0.88 Å (N—H), and with *U*_iso_(H) values set to either 1.2*U*_eq_ or 1.5*U*_eq_ (RCH_3_) of the attached atom.

### Crystal data

**Table:**

C_21_H_17_N_3_O_4_	*Z* = 2
*M**_r_* = 375.38	*F*(000) = 392
Triclinic, *P*1	*D*_x_ = 1.411 Mg m^−^^3^
Hall symbol: -P 1	Mo *K*α radiation, λ = 0.71073 Å
*a* = 7.6390 (1) Å	Cell parameters from 3966 reflections
*b* = 8.0013 (1) Å	θ = 1.0–27.5°
*c* = 15.0405 (3) Å	µ = 0.10 mm^−^^1^
α = 91.9853 (9)°	*T* = 90 K
β = 96.2291 (9)°	Block, colourless
γ = 104.4242 (9)°	0.25 × 0.22 × 0.15 mm
*V* = 883.25 (2) Å^3^	

### Data collection

**Table:**

Nonius KappaCCD diffractometer	3997 independent reflections
Radiation source: fine-focus sealed tube	3595 reflections with *I* > 2σ(*I*)
graphite	*R*_int_ = 0.015
Detector resolution: 18 pixels mm^-1^	θ_max_ = 27.4°, θ_min_ = 1.4°
ω scans at fixed χ = 55°	*h* = −9→9
Absorption correction: multi-scan (*SCALEPACK*; Otwinowski & Minor, 1997)	*k* = −10→10
*T*_min_ = 0.976, *T*_max_ = 0.985	*l* = −19→19
19513 measured reflections	

### Refinement

**Table:**

Refinement on *F*^2^	Secondary atom site location: difference Fourier map
Least-squares matrix: full	Hydrogen site location: inferred from neighbouring sites
*R*[*F*^2^ > 2σ(*F*^2^)] = 0.043	H-atom parameters constrained
*wR*(*F*^2^) = 0.127	*w* = 1/[σ^2^(*F*_o_^2^) + (0.0697*P*)^2^ + 0.3949*P*] where *P* = (*F*_o_^2^ + 2*F*_c_^2^)/3
*S* = 1.07	(Δ/σ)_max_ < 0.001
3997 reflections	Δρ_max_ = 0.48 e Å^−^^3^
255 parameters	Δρ_min_ = −0.34 e Å^−^^3^
0 restraints	Extinction correction: *SHELXL97* (Sheldrick, 2008), Fc^*^=kFc[1+0.001xFc^2^λ^3^/sin(2θ)]^-1/4^
Primary atom site location: structure-invariant direct methods	Extinction coefficient: 0.090 (15)

### Special details

**Table:**

Geometry. All e.s.d.'s (except the e.s.d. in the dihedral angle between two l.s. planes) are estimated using the full covariance matrix. The cell e.s.d.'s are taken into account individually in the estimation of e.s.d.'s in distances, angles and torsion angles; correlations between e.s.d.'s in cell parameters are only used when they are defined by crystal symmetry. An approximate (isotropic) treatment of cell e.s.d.'s is used for estimating e.s.d.'s involving l.s. planes.
Refinement. Refinement of *F*^2^ against ALL reflections. The weighted *R*-factor *wR* and goodness of fit *S* are based on *F*^2^, conventional *R*-factors *R* are based on *F*, with *F* set to zero for negative *F*^2^. The threshold expression of *F*^2^ > 2σ(*F*^2^) is used only for calculating *R*-factors(gt) *etc*. and is not relevant to the choice of reflections for refinement. *R*-factors based on *F*^2^ are statistically about twice as large as those based on *F*, and *R*- factors based on ALL data will be even larger.

### Fractional atomic coordinates and isotropic or equivalent isotropic displacement parameters (Å^2^)

**Table:**

	*x*	*y*	*z*	*U*_iso_*/*U*_eq_	
N1	0.62738 (14)	0.06683 (14)	0.69424 (7)	0.0179 (2)	
C1	0.64933 (17)	0.16626 (17)	0.62219 (8)	0.0189 (3)	
H1	0.7632	0.2298	0.6066	0.023*	
C2	0.44342 (17)	−0.00489 (16)	0.69679 (8)	0.0173 (3)	
C3	0.35580 (18)	−0.11990 (17)	0.75529 (9)	0.0196 (3)	
H3	0.4221	−0.1562	0.8048	0.024*	
C4	0.16793 (19)	−0.17896 (17)	0.73816 (9)	0.0221 (3)	
H4	0.1044	−0.2593	0.7760	0.027*	
C5	0.06985 (18)	−0.12239 (18)	0.66605 (9)	0.0226 (3)	
H5	−0.0589	−0.1643	0.6562	0.027*	
C6	0.15795 (18)	−0.00650 (18)	0.60918 (9)	0.0209 (3)	
H6	0.0904	0.0331	0.5613	0.025*	
C7	0.34801 (17)	0.05157 (16)	0.62314 (8)	0.0177 (3)	
C8	0.48188 (17)	0.16104 (17)	0.57534 (8)	0.0181 (3)	
C9	0.44195 (17)	0.23455 (16)	0.49230 (8)	0.0187 (3)	
H9	0.3177	0.2033	0.4673	0.022*	
C10	0.55628 (18)	0.34163 (16)	0.44489 (8)	0.0186 (3)	
N11	0.74423 (15)	0.41748 (15)	0.46348 (7)	0.0197 (3)	
H11	0.8114	0.4057	0.5131	0.024*	
O12	0.96568 (13)	0.59079 (14)	0.38998 (7)	0.0276 (3)	
C12	0.80892 (18)	0.51160 (16)	0.39495 (9)	0.0198 (3)	
N13	0.66123 (15)	0.50316 (14)	0.33104 (7)	0.0205 (3)	
H13	0.6677	0.5574	0.2811	0.025*	
O14	0.35435 (13)	0.36140 (13)	0.31095 (7)	0.0247 (2)	
C14	0.50269 (18)	0.39906 (16)	0.35539 (8)	0.0191 (3)	
C15	0.77114 (17)	0.06679 (17)	0.76616 (8)	0.0197 (3)	
H15A	0.8908	0.1013	0.7432	0.024*	
H15B	0.7557	−0.0516	0.7868	0.024*	
C16	0.76755 (17)	0.19009 (17)	0.84440 (8)	0.0186 (3)	
C17	0.77627 (19)	0.36332 (18)	0.83052 (9)	0.0230 (3)	
H17	0.7891	0.4045	0.7725	0.028*	
C18	0.76628 (19)	0.47605 (17)	0.90102 (9)	0.0226 (3)	
H18	0.7718	0.5937	0.8910	0.027*	
C19	0.74814 (17)	0.41658 (17)	0.98642 (8)	0.0190 (3)	
C20	0.7411 (2)	0.24417 (18)	1.00064 (9)	0.0244 (3)	
H20	0.7298	0.2033	1.0588	0.029*	
C21	0.7508 (2)	0.13161 (18)	0.92977 (9)	0.0242 (3)	
H21	0.7459	0.0140	0.9398	0.029*	
O22	0.70603 (16)	0.48178 (14)	1.13762 (7)	0.0298 (3)	
C22	0.73352 (17)	0.53113 (17)	1.06416 (9)	0.0196 (3)	
O23	0.75282 (15)	0.69475 (13)	1.04391 (6)	0.0274 (3)	
C23	0.7376 (2)	0.8120 (2)	1.11646 (10)	0.0316 (4)	
H23A	0.6119	0.7839	1.1309	0.047*	
H23B	0.7709	0.9311	1.0982	0.047*	
H23C	0.8197	0.8005	1.1694	0.047*	

### Atomic displacement parameters (Å^2^)

**Table:**

	*U*^11^	*U*^22^	*U*^33^	*U*^12^	*U*^13^	*U*^23^
N1	0.0158 (5)	0.0211 (5)	0.0150 (5)	0.0023 (4)	0.0008 (4)	−0.0007 (4)
C1	0.0186 (6)	0.0219 (6)	0.0150 (6)	0.0023 (5)	0.0034 (4)	−0.0005 (5)
C2	0.0163 (6)	0.0184 (6)	0.0162 (6)	0.0033 (5)	0.0020 (4)	−0.0038 (5)
C3	0.0216 (6)	0.0186 (6)	0.0180 (6)	0.0039 (5)	0.0034 (5)	−0.0005 (5)
C4	0.0230 (7)	0.0205 (6)	0.0219 (6)	0.0018 (5)	0.0077 (5)	−0.0007 (5)
C5	0.0168 (6)	0.0256 (7)	0.0236 (7)	0.0016 (5)	0.0044 (5)	−0.0046 (5)
C6	0.0196 (6)	0.0248 (7)	0.0173 (6)	0.0052 (5)	0.0003 (5)	−0.0022 (5)
C7	0.0189 (6)	0.0196 (6)	0.0141 (6)	0.0040 (5)	0.0029 (4)	−0.0025 (4)
C8	0.0185 (6)	0.0198 (6)	0.0152 (6)	0.0031 (5)	0.0034 (4)	−0.0011 (4)
C9	0.0194 (6)	0.0184 (6)	0.0176 (6)	0.0040 (5)	0.0025 (5)	−0.0010 (5)
C10	0.0202 (6)	0.0176 (6)	0.0176 (6)	0.0047 (5)	0.0018 (5)	−0.0010 (5)
N11	0.0199 (5)	0.0237 (6)	0.0156 (5)	0.0054 (4)	0.0020 (4)	0.0028 (4)
O12	0.0211 (5)	0.0310 (6)	0.0260 (5)	−0.0024 (4)	0.0022 (4)	0.0074 (4)
C12	0.0234 (6)	0.0173 (6)	0.0177 (6)	0.0032 (5)	0.0021 (5)	0.0006 (5)
N13	0.0232 (6)	0.0196 (5)	0.0172 (5)	0.0025 (4)	0.0013 (4)	0.0039 (4)
O14	0.0234 (5)	0.0281 (5)	0.0219 (5)	0.0058 (4)	−0.0005 (4)	0.0062 (4)
C14	0.0222 (6)	0.0180 (6)	0.0181 (6)	0.0066 (5)	0.0036 (5)	0.0011 (5)
C15	0.0170 (6)	0.0242 (6)	0.0174 (6)	0.0056 (5)	−0.0011 (5)	−0.0013 (5)
C16	0.0151 (6)	0.0222 (6)	0.0174 (6)	0.0041 (5)	−0.0008 (4)	−0.0009 (5)
C17	0.0283 (7)	0.0230 (7)	0.0158 (6)	0.0032 (5)	0.0021 (5)	0.0024 (5)
C18	0.0300 (7)	0.0185 (6)	0.0180 (6)	0.0037 (5)	0.0018 (5)	0.0021 (5)
C19	0.0187 (6)	0.0206 (6)	0.0166 (6)	0.0038 (5)	0.0000 (5)	−0.0001 (5)
C20	0.0336 (8)	0.0239 (7)	0.0167 (6)	0.0089 (6)	0.0033 (5)	0.0037 (5)
C21	0.0330 (7)	0.0202 (6)	0.0205 (6)	0.0091 (5)	0.0023 (5)	0.0024 (5)
O22	0.0460 (7)	0.0272 (5)	0.0174 (5)	0.0101 (5)	0.0075 (4)	0.0024 (4)
C22	0.0182 (6)	0.0214 (6)	0.0180 (6)	0.0039 (5)	−0.0010 (5)	0.0012 (5)
O23	0.0444 (6)	0.0197 (5)	0.0185 (5)	0.0090 (4)	0.0046 (4)	−0.0005 (4)
C23	0.0490 (10)	0.0245 (7)	0.0224 (7)	0.0130 (7)	0.0023 (6)	−0.0047 (6)

### Geometric parameters (Å, °)

**Table:**

N1—C1	1.3668 (17)	C12—N13	1.3869 (17)
N1—C2	1.3846 (16)	N13—C14	1.3819 (17)
N1—C15	1.4554 (16)	N13—H13	0.8800
C1—C8	1.3821 (18)	O14—C14	1.2137 (16)
C1—H1	0.9500	C15—C16	1.5162 (17)
C2—C3	1.3951 (18)	C15—H15A	0.9900
C2—C7	1.4093 (18)	C15—H15B	0.9900
C3—C4	1.3866 (19)	C16—C21	1.3898 (18)
C3—H3	0.9500	C16—C17	1.3951 (19)
C4—C5	1.403 (2)	C17—C18	1.3890 (19)
C4—H4	0.9500	C17—H17	0.9500
C5—C6	1.3833 (19)	C18—C19	1.3940 (18)
C5—H5	0.9500	C18—H18	0.9500
C6—C7	1.3996 (18)	C19—C20	1.3921 (19)
C6—H6	0.9500	C19—C22	1.4916 (18)
C7—C8	1.4445 (17)	C20—C21	1.3915 (19)
C8—C9	1.4340 (18)	C20—H20	0.9500
C9—C10	1.3454 (18)	C21—H21	0.9500
C9—H9	0.9500	O22—C22	1.2083 (16)
C10—N11	1.4053 (17)	C22—O23	1.3303 (16)
C10—C14	1.4858 (18)	O23—C23	1.4475 (16)
N11—C12	1.3612 (17)	C23—H23A	0.9800
N11—H11	0.8800	C23—H23B	0.9800
O12—C12	1.2189 (17)	C23—H23C	0.9800
			
C1—N1—C2	109.12 (11)	C14—N13—H13	124.2
C1—N1—C15	124.49 (11)	C12—N13—H13	124.2
C2—N1—C15	125.24 (11)	O14—C14—N13	126.35 (12)
N1—C1—C8	110.17 (11)	O14—C14—C10	128.62 (12)
N1—C1—H1	124.9	N13—C14—C10	105.00 (11)
C8—C1—H1	124.9	N1—C15—C16	111.22 (10)
N1—C2—C3	129.95 (12)	N1—C15—H15A	109.4
N1—C2—C7	107.54 (11)	C16—C15—H15A	109.4
C3—C2—C7	122.43 (12)	N1—C15—H15B	109.4
C4—C3—C2	117.24 (12)	C16—C15—H15B	109.4
C4—C3—H3	121.4	H15A—C15—H15B	108.0
C2—C3—H3	121.4	C21—C16—C17	119.35 (12)
C3—C4—C5	121.30 (12)	C21—C16—C15	120.72 (12)
C3—C4—H4	119.3	C17—C16—C15	119.91 (11)
C5—C4—H4	119.3	C18—C17—C16	120.43 (12)
C6—C5—C4	120.95 (12)	C18—C17—H17	119.8
C6—C5—H5	119.5	C16—C17—H17	119.8
C4—C5—H5	119.5	C17—C18—C19	120.04 (12)
C5—C6—C7	119.10 (12)	C17—C18—H18	120.0
C5—C6—H6	120.5	C19—C18—H18	120.0
C7—C6—H6	120.5	C20—C19—C18	119.66 (12)
C6—C7—C2	118.93 (12)	C20—C19—C22	118.03 (12)
C6—C7—C8	133.86 (12)	C18—C19—C22	122.31 (12)
C2—C7—C8	107.19 (11)	C21—C20—C19	120.11 (12)
C1—C8—C9	128.94 (12)	C21—C20—H20	119.9
C1—C8—C7	105.98 (11)	C19—C20—H20	119.9
C9—C8—C7	124.90 (12)	C16—C21—C20	120.42 (13)
C10—C9—C8	128.91 (12)	C16—C21—H21	119.8
C10—C9—H9	115.5	C20—C21—H21	119.8
C8—C9—H9	115.5	O22—C22—O23	123.20 (12)
C9—C10—N11	130.48 (12)	O22—C22—C19	124.14 (12)
C9—C10—C14	124.45 (12)	O23—C22—C19	112.66 (11)
N11—C10—C14	105.04 (11)	C22—O23—C23	115.26 (11)
C12—N11—C10	111.28 (11)	O23—C23—H23A	109.5
C12—N11—H11	124.4	O23—C23—H23B	109.5
C10—N11—H11	124.4	H23A—C23—H23B	109.5
O12—C12—N11	127.32 (12)	O23—C23—H23C	109.5
O12—C12—N13	125.72 (12)	H23A—C23—H23C	109.5
N11—C12—N13	106.95 (11)	H23B—C23—H23C	109.5
C14—N13—C12	111.67 (11)		
			
C2—N1—C1—C8	−0.64 (14)	C10—N11—C12—N13	1.94 (14)
C15—N1—C1—C8	−168.87 (11)	O12—C12—N13—C14	178.35 (13)
C1—N1—C2—C3	177.11 (13)	N11—C12—N13—C14	−2.48 (15)
C15—N1—C2—C3	−14.8 (2)	C12—N13—C14—O14	−176.40 (13)
C1—N1—C2—C7	0.36 (14)	C12—N13—C14—C10	1.99 (14)
C15—N1—C2—C7	168.48 (11)	C9—C10—C14—O14	−0.3 (2)
N1—C2—C3—C4	−176.00 (12)	N11—C10—C14—O14	177.59 (13)
C7—C2—C3—C4	0.32 (18)	C9—C10—C14—N13	−178.67 (12)
C2—C3—C4—C5	−1.33 (19)	N11—C10—C14—N13	−0.75 (13)
C3—C4—C5—C6	0.5 (2)	C1—N1—C15—C16	94.54 (14)
C4—C5—C6—C7	1.38 (19)	C2—N1—C15—C16	−71.81 (15)
C5—C6—C7—C2	−2.33 (18)	N1—C15—C16—C21	122.96 (13)
C5—C6—C7—C8	175.72 (13)	N1—C15—C16—C17	−55.33 (16)
N1—C2—C7—C6	178.56 (11)	C21—C16—C17—C18	−0.7 (2)
C3—C2—C7—C6	1.52 (18)	C15—C16—C17—C18	177.60 (12)
N1—C2—C7—C8	0.03 (14)	C16—C17—C18—C19	0.3 (2)
C3—C2—C7—C8	−177.01 (11)	C17—C18—C19—C20	0.4 (2)
N1—C1—C8—C9	−174.63 (12)	C17—C18—C19—C22	−178.91 (12)
N1—C1—C8—C7	0.63 (14)	C18—C19—C20—C21	−0.5 (2)
C6—C7—C8—C1	−178.62 (14)	C22—C19—C20—C21	178.79 (12)
C2—C7—C8—C1	−0.40 (14)	C17—C16—C21—C20	0.6 (2)
C6—C7—C8—C9	−3.1 (2)	C15—C16—C21—C20	−177.73 (12)
C2—C7—C8—C9	175.11 (12)	C19—C20—C21—C16	0.0 (2)
C1—C8—C9—C10	−7.9 (2)	C20—C19—C22—O22	−4.2 (2)
C7—C8—C9—C10	177.63 (13)	C18—C19—C22—O22	175.06 (13)
C8—C9—C10—N11	−2.6 (2)	C20—C19—C22—O23	176.23 (12)
C8—C9—C10—C14	174.74 (12)	C18—C19—C22—O23	−4.50 (18)
C9—C10—N11—C12	177.01 (13)	O22—C22—O23—C23	−0.3 (2)
C14—C10—N11—C12	−0.74 (14)	C19—C22—O23—C23	179.24 (11)
C10—N11—C12—O12	−178.91 (13)		

### Hydrogen-bond geometry (Å, °)

**Table:**

*D*—H···*A*	*D*—H	H···*A*	*D*···*A*	*D*—H···*A*
N11—H11···O12^i^	0.88	2.11	2.9658 (15)	163
N13—H13···O22^ii^	0.88	2.29	2.9699 (15)	134

Symmetry codes: (i) −*x*+2, −*y*+1, −*z*+1; (ii) *x*, *y*, *z*−1.

## References

[bb1] Mason, M. R., Barnard, T. S., Segla, M. F., Xie, B. & Kirschbaum, K. (2003). *J. Chem. Crystallogr.* **33**, 531–540.

[bb2] Nonius (1998). *COLLECT* Nonius BV, Delft, The Netherlands.

[bb3] Otwinowski, Z. & Minor, W. (1997). *Methods in Enzymology*, Vol. 276, *Macromolecular Crystallography*, Part A, edited by C. W. Carter Jr & R. M. Sweet, pp. 307–326. New York: Academic Press.

[bb4] Sekhar, K. R., Crooks, P. A., Sonar, V. N., Friedman, D. B., Chan, J. Y., Meredith, M. J., Stames, J. H., Kelton, K. R., Summar, S. R., Sasi, S. & Freeman, M. L. (2003). *Cancer Res.* **63**, 5636–5645. 14500406

[bb5] Sheldrick, G. M. (2008). *Acta Cryst.* A**64**, 112–122. 10.1107/S0108767307043930 18156677

[bb6] Sonar, V. N., Reddy, Y. T., Sekhar, K. R., Sowmya, S., Freeman, M. L. & Crooks, P. A. (2007). *Bioorg. Med. Chem. Lett.* **17**, 6821–6824. 10.1016/j.bmcl.2007.10.035 PMC3726020 17980582

[bb7] Zarza, P. M., Gill, P., Díaz González, M. C., Martin Reyes, M. G., Arrieta, J. M., Nastopoulos, V., Germain, G. & Debaerdemaeker, T. (1988). *Acta Cryst.* C**44**, 678–681.

